# The mitochondrial profile in women with polycystic ovary syndrome: impact of exercise

**DOI:** 10.1530/JME-21-0177

**Published:** 2022-01-21

**Authors:** Melpomeni Malamouli, Itamar Levinger, Andrew J McAinch, Adam J Trewin, Raymond J Rodgers, Alba Moreno-Asso

**Affiliations:** 1 Institute for Health and Sport (iHeS), Victoria University, Melbourne, Victoria, Australia; 2 Australian Institute for Musculoskeletal Science (AIMSS), Western Health, Victoria University, Victoria, Australia; 3 Institute for Physical Activity and Nutrition, Deakin University, Geelong, Victoria, Australia; 4 The Robinson Research Institute, School of Medicine, University of Adelaide, Adelaide, South Australia, Australia

**Keywords:** polycystic ovary syndrome, mitochondria, insulin resistance, exercise, metabolic health

## Abstract

Polycystic ovary syndrome (PCOS) is a common endocrine disorder affecting pre-menopausal women and involves metabolic dysregulation. Despite the high prevalence of insulin resistance, the existence of mitochondrial dysregulation and its role in the pathogenesis of PCOS is not clear. Exercise is recommended as the first-line therapy for women with PCOS. In particular, high-intensity interval training (HIIT) is known to improve metabolic health and enhance mitochondrial characteristics. In this narrative review, the existing knowledge of mitochondrial characteristics in skeletal muscle and adipose tissue of women with PCOS and the effect of exercise interventions in ameliorating metabolic and mitochondrial health in these women are discussed. Even though the evidence on mitochondrial dysfunction in PCOS is limited, some studies point to aberrant mitochondrial functions mostly in skeletal muscle, while there is very little research in adipose tissue. Although most exercise intervention studies in PCOS report improvements in metabolic health, they show diverse and inconclusive findings in relation to mitochondrial characteristics. A limitation of the current study is the lack of comprehensive mitochondrial analyses and the diversity in exercise modalities, with only one study investigating the impact of HIIT alone. Therefore, further comprehensive large-scale exercise intervention studies are required to understand the association between metabolic dysfunction and aberrant mitochondrial profile, and the molecular mechanisms underlying the exercise-induced metabolic adaptations in women with PCOS.

## Introduction

Polycystic ovary syndrome (PCOS) is a widely under-diagnosed syndrome affecting reproductive and metabolic health in women of reproductive age (Teede *et al.* 2010). The prevalence of PCOS varies between 8 and 15% of women worldwide depending on the diagnostic criteria used and the population studied (Bozdag *et al.* 2016). PCOS is characterised by a hormonal imbalance with increased levels of luteinising hormone (LH) and androgen excess, leading to irregular menstrual cycles, anovulation and perpetuating hyperandrogenism (Bozdag *et al.* 2016). Women with PCOS present with health manifestations across the lifespan, causing a major economic and health burden (Teede *et al.* 2010). Currently, the most widely internationally recognised criteria for diagnosing PCOS are the Rotterdam criteria, which require any two of the following features to be fulfilled: (1) oligo- or anovulation, (2) clinical or biochemical hyperandrogenism, and (3) polycystic ovaries on ultrasound (Rotterdam ESHRE/ASRM-Sponsored PCOS Consensus Workshop Group 2004). Insulin resistance (IR) is also an important metabolic hallmark of PCOS, leading to compensatory hyperinsulinaemia and metabolic dysfunction (Teede *et al.* 2010). IR is present in 38–95% of women with PCOS, and is aggravated by, but independent of obesity (Stepto *et al.* 2013). Other metabolic features present in women with PCOS include compensatory hyperinsulinaemia and associated risk of type 2 diabetes mellitus (TDM2), gestational diabetes (GDM), impaired glucose tolerance, dyslipidemia and increased risk factors for cardiovascular disease (Rodgers *et al.* 2019).

Mitochondria, despite being the main organelles responsible for cellular energy production, have received limited investigation in women with PCOS. However, in other metabolic and cardiovascular disorders, mitochondria are reported to be dysregulated (Patti & Corvera 2010). In obesity and T2DM, mitochondrial dysfunction has been associated with IR (Das *et al.* 2021). To date, two proposed mechanisms implicate mitochondrial dysfunction in IR: (1) incomplete oxidation of fatty acids, resulting in lipid accumulation, which may inhibit insulin signalling, and (2) impaired substrate oxidation causing increased reactive oxygen species (ROS) and oxidative stress, potentially resulting in mitophagy and apoptosis (Schmitz-Peiffer *et al.* 1999, Fan *et al.* 2019). This could lead to decreased substrate oxidation, further aggravating lipid accumulation. However, mitochondrial dysfunction does not always imply IR and vice versa (Irving *et al.* 2011).

Mitochondrial dysfunction has been associated with PCOS-specific IR in some animal and human studies, yet, the aetiology of this association and whether mitochondrial dysfunction has a direct effect on the pathogenesis of PCOS is unclear. The majority of studies investigating mitochondrial profile in PCOS have been done in ovaries, liver, skeletal muscle and blood (Shukla & Mukherjee 2020).

Skeletal muscle plays an important role in whole-body glucose regulation. Defects in skeletal muscle insulin signalling contribute to insulin-resistant conditions including PCOS (Stepto *et al.* 2019). However, whether this impaired insulin signalling in the skeletal muscle may be linked with mitochondrial dysregulation remains to be determined. Adipose tissue plays a wide-ranging role in metabolic regulation and physiological homeostasis, and its excess is associated with IR, T2DM, hypertension and cardiovascular disease. Women with PCOS often have a higher amount of visceral fat than women without PCOS, which exacerbates IR (Cascella *et al.* 2008). However, whether the adipose tissue abnormalities are primary or secondary to mitochondrial dysfunction in adipocytes is yet to be determined.

Lifestyle interventions, such as exercise, have been used to target the metabolic and reproductive imbalances associated with PCOS. Exercise remains the first-line treatment to manage the symptoms and improve the clinical features of PCOS, including increased insulin sensitivity, cardiorespiratory fitness, menstrual cyclicity and ovulation, reduced body weight, waist-to-hip ratio, waist circumference, total testosterone, hirsutism, and improved mental health (Moran *et al.* 2011, Teede *et al.* 2018). Exercise can also improve mitochondrial function. In particular, high-intensity interval training (HIIT) seems to result in larger mitochondrial health benefits in different population groups when compared with moderate-intensity exercise interventions, and it has also been found to lead to greater health outcomes in women with PCOS (Patten *et al.* 2020).

This review will summarise the findings on mitochondrial characteristics such as mtDNA content, mitochondrial respiration and oxidative phosphorylation (OXPHOS), mitochondrial dynamics and ROS production in the skeletal muscle and adipose tissue of women with PCOS and will also discuss the effect of different training exercise modalities on metabolic and mitochondrial features of women with PCOS.

## Characteristics of mitochondrial dysregulation

**Figure 1:**
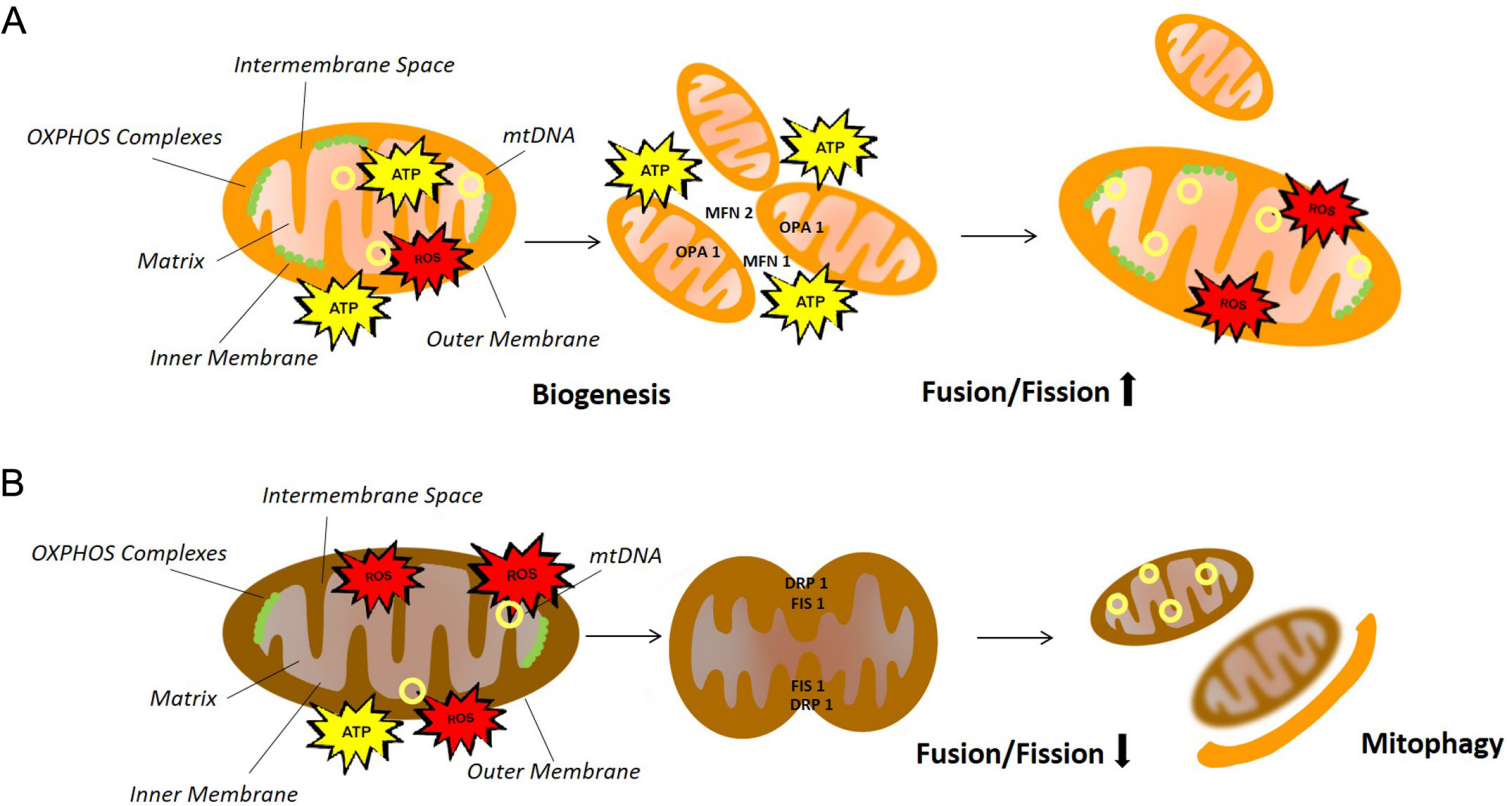
Healthy mitochondria vs dysfunctional mitochondria. (A) Schematic representation of regulated functions of mitochondria including sufficient ATP synthesis, balanced levels of ROS production, biogenesis and increased fusion/fission ratio. (B) Schematic representation of the mechanisms causing dysfunctional mitochondria, including defects in activity of oxidative phosphorylation (OXPHOS) complexes, reduced mitochondrial respiration, increased reactive oxygen species (ROS) production, abnormalities in mitochondrial DNA (mtDNA) copy number, reduced mitochondrial biogenesis and dysregulated fusion/fission ratio and mitophagy.

Mitochondria have numerous critical roles in metabolism as the main site of oxidation of substrates derived from glucose, fatty acids and amino acids. Mitochondria are also a major player in 1-carbon metabolism and nitrogen metabolism, as well as essential in the steroid biosynthesis, synthesis of haem and iron–sulphur clusters. Mitochondria can mediate intracellular signaling by the production of ROS and interact with other organelles such as the endoplasmic reticulum (ER). The ER–mitochondrial communication regulates mitochondrial activity and is vital for maintaining intracellular calcium homeostasis, as well as metabolic processes such as phospholipid metabolism, which relies on lipid translocation between ER and mitochondria. However, their most prominent role is energy production in the form of ATP via aerobic respiration. Aerobic respiration involves glycolysis, citric acid cycle and OXPHOS (Spinelli & Haigis 2018). Mitochondrial dysfunction manifests as an inability to sustain ATP synthesis sufficiently to satisfy cellular energy demands. This can result not only from reduced mitochondrial respiration but also from mtDNA mutations, abnormalities in mtDNA copy number, defects in activity of OXPHOS complexes, reduced mitochondrial biogenesis, dysregulated mitophagy and dysregulation of other processes such as calcium handling or aberrant production of ROS leading to oxidative stress (Heilbronn *et al.* 2007) (Fig. 1). Therefore, mitochondrial dysfunction is a broad term that can include impairments in several mitochondrial characteristics.

### Mitochondrial content

Mitochondrial content represents the amount of all mitochondrial constituents within a cell. However, because it is not feasible to measure all of these molecules, quantifiable markers commonly used to correlate well with mitochondrial volume are assessed by the gold-standard transmission electron microscopy (Larsen *et al.* 2012). mtDNA copy number is the most common mitochondrial content marker reported in the literature and is used as an indicative marker of mitochondrial biogenesis, which is thought to augment upon increased energy demands, such as exercise (Holloszy & Booth 1976), and also as a compensatory mechanism for mitochondrial dysfunction (Filograna *et al.* 2021). Lower mtDNA copy number have been associated with increased risk of T2DM and metabolic syndrome in large-scale human studies (Fazzini *et al.* 2021).

Most of the current studies have measured mtDNA content in blood of women with PCOS, with limited research conducted in muscle and adipose tissue (Table 1). Findings in skeletal muscle of women with PCOS showed no alterations in mtDNA copy number or other markers of mitochondrial content, citrate synthase (CS) and fatty acid oxidation indicated by hydroxyacyl-CoA dehydrogenase subunit beta (β-HAD) activity (Hutchison *et al.* 2011, Rabøl *et al.* 2011, Konopka *et al.* 2015) (Table 1). Similarly, no alterations in mitochondrial mass were detected in primary myotubes of insulin-resistant women with PCOS compared to healthy women using the MitoTracker Green FM probe (Eriksen *et al.* 2011). Therefore, current evidence suggests that mitochondrial content is not altered in skeletal muscle of women with PCOS, but further research using the gold-standard electron microscopy for the analysis of mitochondrial content is required to confirm these findings.

**Table 1:** Studies of mitochondrial content.

Mitochondrial content assessment	Population	Tissue		Study
		Skeletal muscle	Adipose tissue	
Mitochondrial DNA copy number	23 PCOS (9 lean and 14 obese) 17 age- and weight-matched ctrls (6 lean and 11 obese)	↔	–	(Rabøl *et al.* 2011)
Mitochondrial mass by MitoTracker Green Probe	8 insulin-resistant PCOS 8 weight- and age-matched healthy ctrls	↔ (cultured primary myotubes)	–	(Eriksen *et al.* 2011)
Citrate synthase activity	16 overweight and obese PCOS 13 fat mass- and BMI-matched ctrls	↔	–	(Hutchison *et al.* 2012)
	25 obese insulin-resistant PCOS 14 lean insulin-sensitive ctrls	↔	–	(Konopka *et al.* 2015)
	9 lean insulin-sensitive PCOS 9 age- and BMI-matched ctrls	↔	–	(Hansen *et al.* 2020)

(↔), no differences; (–), not measured; PCOS, women with PCOS, ctrls, control women.

The mitochondrial content in adipose tissue of women with PCOS has not been examined yet, and therefore, it would be a valuable addition to this area of research. Evidence from people who are obese suggests that decreased mtDNA copy number in the adipose tissue is associated with higher BMI (Fischer *et al.* 2015), but whether there is an association with PCOS, independent of BMI, is unknown. In addition, no study to date has compared the mtDNA copy number between skeletal muscle and adipose tissue in women with PCOS to elucidate whether these abnormalities may be tissue-specific or distinctive of PCOS pathogenesis.

### Mitochondrial respiration: OXPHOS

OXPHOS involves the transfer of electrons through the mitochondrial electron transport chain (ETC) complexes I–IV, while simultaneously pumping protons to the intermembrane space. This proton gradient generates a membrane potential that is utilized by ATP synthase (complex V) to drive phosphorylation of ADP to ATP.

To date, there are conflicting results in regards to OXPHOS genes expression in women with PCOS (Table 2). It was firstly reported that the OXPHOS genes *NDUFA3, SDHD, UCRC, COX7C* and *ATP5H* are downregulated in the five respiratory complexes in the skeletal muscle of obese insulin-resistant women with PCOS compared to BMI-matched healthy controls (Skov *et al.* 2007). In contrast, Hutchison *et al.* found no differences in skeletal muscle OXPHOS gene expression and protein abundance between overweight and obese women with PCOS and fat mass- and BMI-matched women (Hutchison *et al.* 2012). In line with that, no differences were seen either in skeletal muscle phosphorylating and uncoupled respiration between obese insulin-resistant women with PCOS and lean insulin-sensitive women without PCOS (Konopka *et al.* 2015). However, mitochondrial respiration during leak/state 4 (the rate of oxygen consumption in the absence of ADP) was increased in obese women with PCOS, consistent with a decrease in mitochondrial coupling efficiency (Konopka *et al.* 2015). In primary myotubes derived from insulin-resistant women with PCOS, no differences were observed in mitochondrial respiration for complex I and complex II and in oxidation of glucose and ATP synthesis compared to myotubes from weight- and age-matched healthy women (Eriksen *et al.* 2011, Rabøl *et al.* 2011). The only study of mitochondrial respiration in adipose tissue showed decreased maximal oxygen flux in subcutaneous abdominal adipose tissue in women with PCOS but no difference in the gluteal adipose tissue of these women compared to controls (Lionett *et al.* 2021).

**Table 2:** Studies of mitochondrial respiration: OXPHOS.

Mitochondrial respiration: OXPHOS assessment	Population	Tissue		Study
		Skeletal muscle	Adipose tissue	
OXPHOS gene expression	16 obese insulin-resistant PCOS 13 age- and BMI-matched healthy ctrls	↓	–	(Skov *et al.* 2007)
	16 overweight and obese PCOS 13 fat mass- and BMI-matched ctrls	↔	–	(Hutchison *et al.* 2012)
OXPHOS protein abundance	16 overweight and obese PCOS 13 fat mass- and BMI-matched ctrls	↔	–	(Hutchison *et al.* 2012)
ATP synthesis	8 insulin-resistant PCOS 8 weight- and age-matched healthy ctrls	↔ (myotubes)	–	(Eriksen *et al.* 2011)
Respiration (state 3)	23 PCOS (9 lean and 14 obese) 17 age- and weight-matched ctrls (6 lean and 11 obese)	↔	–	(Rabøl *et al.* 2011)
	25 obese insulin-resistant PCOS 14 lean insulin-sensitive ctrls	↔	–	(Konopka *et al.* 2015)
	18 PCOS 15 age- and BMI-matched ctrls	–	↔ (subcutaneous gluteal)	(Lionett *et al.* 2021)
	16 PCOS 10 age- and BMI-matched ctrls	–	↓ (subcutaneous abdominal)	(Lionett *et al.* 2021)
Respiration (state 4)	25 obese insulin-resistant PCOS 14 lean insulin-sensitive ctrls	↑	–	(Konopka *et al.* 2015)
Uncoupling control ratio (electron transport capacity)	23 PCOS (9 lean and 14 obese) 17 age- and weight-matched ctrls (6 lean and 11 obese)	↑ (in obese controls than any other group)	–	(Rabøl *et al.* 2011)
Uncoupled respiration (maximal electron flux capacity)	14 obese PCOS 11 age- and weight-matched ctrls	↓	–	(Rabøl *et al.* 2011)
	9 lean PCOS 6 age- and weight-matched ctrls	↔	–	(Rabøl *et al.* 2011)
	25 obese insulin-resistant PCOS 14 lean insulin-sensitive ctrls	↔	–	(Konopka *et al.* 2015)
Phosphorylation efficiency (ADP:O)	25 obese insulin-resistant PCOS 14 lean insulin-sensitive ctrls	↓	–	(Konopka *et al.* 2015)
Coupling efficiency	25 obese insulin-resistant PCOS 14 lean insulin-sensitive ctrls	↓	–	(Konopka *et al.* 2015)

(↔), no differences; (↓), downregulation; (↑), upregulation; (–), not measured; PCOS, women with PCOS; ctrls, control women.

Altogether, the findings reported within each tissue are equivocal, with inconsistent findings also reported across skeletal muscle and adipose tissue of women with PCOS. Therefore, there is a need for further validation studies to elucidate whether impaired mitochondrial function is indeed existent in PCOS and whether it is tissue specific.

### Mitochondrial dynamics

Mitochondrial dynamics involve the dynamic processes of mitochondrial biogenesis, fusion, fission and mitophagy (Ferree & Shirihai 2012). Biogenesis is the process through which mitochondria increase their mass. Peroxisome proliferator-activated receptor gamma coactivator-1 alpha (PGC1A), a transcriptional coactivator, is a major regulator of the mitochondrial biogenic programme (Heilbronn *et al.* 2007). PGC1A interacts with nuclear respiratory factor 1 (NRF1), stimulating transcription of many nuclear-encoded mitochondrial genes including mitochondrial transcription factor A (*TFAM*), a direct regulator of mitochondrial DNA replication and transcription (Heilbronn *et al.* 2007). Mitochondria undergo fission and fusion (elongation) to adapt to changes in the cellular environment (Suárez-Rivero *et al.* 2017). Mitochondrial fusion produces tubular or elongated mitochondria which allows exchanging of material between mitochondria and may compensate for functional defects (Suárez-Rivero *et al.* 2017). In contrast, mitochondrial fission is needed to create new mitochondria, but it also allows the segregation of damaged mitochondria (Suárez-Rivero *et al.* 2017). Suboptimal or damaged mitochondria can be either eliminated by mitophagy, a mechanism of selective elimination of damaged mitochondria, or be fused with healthy mitochondria to increase their size and activity (Diaz-Vegas *et al.* 2020). Biogenesis and mitophagy are two opposing mechanisms but essential to maintain mitochondrial and cellular homeostasis.

Mitochondrial biogenesis has only been assessed in a few studies in women with PCOS, mainly in skeletal muscle (Table 3). Expression of *PGC1A* gene was found to be significantly downregulated in the skeletal muscle of obese insulin-resistant women with PCOS, compared to BMI- and age-matched healthy women (Skov *et al.* 2007). This reduction in *PGC1A* was accompanied by a decreased expression of OXPHOS genes but unaltered gene expression of the other mitochondrial biogenesis markers *PGC1B* and *NRF1*(Skov *et al.* 2007). However, another study did not find differences in *PGC1A*, nuclear respiratory factor (*NRF1*) and *TFAM* gene expression between skeletal muscle of overweight and obese women with or without PCOS (Hutchison *et al.* 2012), failing to support the previous findings. No studies on mitochondrial biogenesis in women with PCOS have been reported in adipose tissue.

**Table 3:** Studies of mitochondrial dynamics.

Mitochondrial dynamics assessment	Population	Tissue		Study
		Skeletal muscle	Adipose tissue	
*PGC1A* gene expression	16 obese insulin-resistant PCOS 13 age- and BMI-matched healthy ctrls	↓	–	(Skov *et al.* 2007)
	4 obese PCOS women 4 body composition-matched controls	↑	–	(Dantas *et al.* 2017)
*PGC1A* gene expression and protein abundance	16 overweight and obese PCOS 13 fat mass- and BMI-matched ctrls	↔	–	(Hutchison *et al.* 2012)
*PGC1B* gene expression	16 obese insulin-resistant PCOS 13 age- and BMI-matched healthy ctrls	↔	–	(Skov *et al.* 2007)
NRF1 gene expression		↔	–	(Skov *et al.* 2007)
TFAM gene expression	16 overweight and obese PCOS 13 fat mass- and BMI-matched ctrls	↔	–	(Hutchison *et al.* 2012)

(↔), no differences; (↓), downregulation; (↑), upregulation; (–), not measured; PCOS, women with PCOS; ctrls, control women.

To date, mitophagy has only been assessed in ovarian tissue and has not been investigated in skeletal muscle or adipose tissue of women with PCOS. Fusion and fission are yet to be explored in any tissue in PCOS, despite dysregulation of these events having been previously associated with IR in skeletal muscle (Jheng *et al.* 2012). It is hypothesised that fusion is associated with higher metabolic activity while increased fission is usually noted in metabolic disease (Babbar & Saeed Sheikh 2013). However, whether this happens in PCOS is currently unknown. Therefore, there is a need to uncover the mitochondrial dynamics in metabolic tissues such as skeletal muscle and adipose tissue of women with PCOS and identify any association with the pathogenesis of this syndrome.

### ROS production

Mitochondria not only play a central role in metabolism through ATP production but also in intracellular signalling necessary for the whole cell function (Chandel 2015). Production of ROS is one of the mechanisms by which mitochondria activate transcription factors to regulate intracellular signalling (Chandel 2015). Mitochondria are the main site of ROS production, as single electrons can leak from a number of sites of the ETC and react with oxygen, producing superoxide anion (O_2_^·^) which is the primary form of ROS. ROS include other chemically reactive molecules containing molecular oxygen, such as singlet oxygen (_1_O_2_), hydrogen peroxide (H_2_O_2_) and hydroxyl radicals (^·^OH) (Bayir 2005). At appropriate levels, ROS production is important for cellular signalling, gene expression, metabolic regulation and immune responses. However, excessive ROS formation can lead to oxidative stress, which is defined as an imbalance between oxidative and anti-oxidative systems of cells and tissues causing the disturbance of redox pathways and molecular damage (Jones 2006).

Excessive mitochondrial ROS production and oxidative stress can induce mitochondrial dysfunction that may contribute to the pathology of many metabolic and cardiovascular diseases, such as T2DM, atherosclerosis, obesity and IR (Das *et al.* 2021). Similarly, there is evidence that increased ROS may play a role in the pathogenesis of PCOS (Uyanikoglu *et al.* 2013).

Only one study has been conducted in skeletal muscle of women with PCOS, while no studies have explored ROS production in the adipose tissue (Table 4). In skeletal muscle, mitochondrial hydrogen peroxide (mtH_2_O_2_) emissions assessed by Amplex Red fluorescence has been found to be significantly higher in obese, insulin-resistant women with PCOS, supporting the relationship between mtH_2_O_2_ emissions and IR in this tissue (Konopka *et al.* 2015). These findings, however, may be associated with obesity as skeletal muscle mtH_2_O_2_ emissions have also been shown in obesity-induced IR (Fisher-Wellman *et al.* 2014), and therefore, this might be a major contributor to mitochondrial oxidative stress in PCOS, exacerbating the PCOS-specific IR. Despite an association between increased ROS levels and obesity-induced adipose tissue inflammation and IR (Han 2016), ROS levels have never been investigated in adipose tissue of women with PCOS. Altogether, there is a need for further studies in skeletal muscle and to explore whether there exists a PCOS-specific increase in ROS production in adipose tissue, independent of obesity.

**Table 4:** Studies of ROS production.

ROS production assessment	Population	Tissue		Study
		Skeletal muscle	Adipose tissue	
Mitochondrial H_2_O_2_ emissions	25 obese insulin-resistant PCOS 14 lean insulin-sensitive ctrls	↑	–	(Konopka *et al.* 2015)

(↑), upregulation; (–), not measured; PCOS, women with PCOS; ctrls, control women.

## Effect of exercise on mitochondrial characteristics in PCOS

### Exercise intervention in PCOS

Regular exercise is regarded as a first-line therapy of lifestyle modification in women with PCOS (Teede *et al.* 2018). Exercise training induces a multitude of positive, health-related outcomes in women with PCOS including reproductive, metabolic and mental health benefits (Hutchison *et al.* 2011, Barber *et al.* 2019). These improvements are associated with increased cardiorespiratory fitness (VO_2peak_), decreased waist circumference and improvement in various markers of metabolic health, including insulin sensitivity as measured by euglycaemic–hyperinsulinaemic clamp, fasting insulin and homeostatic model assessment of insulin resistance (HOMA-IR) (Patten *et al.* 2020). The international evidence-based guidelines for the assessment and management of PCOS recommends 150 min/week of moderate intensity or 75 min/week of vigorous intensity exercise for all women with PCOS (Teede *et al.* 2018).

Recent evidence suggests that HIIT, which comprises repeated, short bouts of high-intensity exercise interspersed with rest periods, may have more beneficial metabolic outcomes in comparison to other exercise modalities with lower intensities in women with PCOS. Specifically, an improvement in HOMA-IR and body composition was observed after 10 weeks of HIIT with lean and overweight women with PCOS (Almenning *et al.* 2015). In line with that, a cross-sectional study of 326 women with PCOS showed that vigorous exercise resulted in lower BMI and HOMA-IR compared to moderate exercise (Greenwood *et al.* 2016). In the latter study, 60 min of vigorous intensity exercise per week but not moderate intensity exercise was associated with a 22% reduction in odds of developing metabolic syndrome independent of age, BMI and total energy expenditure (Greenwood *et al.* 2016). In addition, a 16% improvement in insulin sensitivity as determined by euglycaemic–hyperinsulinaemic clamp was observed in women with PCOS following a 12-week vigorous intensity exercise intervention (Harrison *et al.* 2012).

Despite the beneficial health impact of vigorous exercise on women with PCOS, there is a gap in the knowledge about the underlying exercise-induced mechanisms associated with improvements in metabolic outcomes and whether the mitochondrial adaptations are related to these clinical changes.

### Exercise-induced changes on mitochondrial characteristics in PCOS

It is well established that exercise increases mitochondrial biogenesis, mitochondrial content and mitochondrial function. However, HIIT in particular has been shown to provide greater mitochondrial-related benefits compared to other exercise training modalities (Robinson *et al.* 2018). It has been previously shown that HIIT increases skeletal muscle oxidative capacity in obese insulin-resistant patients, independent of obesity (De Matos *et al.* 2018). These improved mitochondrial characteristics were accompanied by a reduction in HOMA-IR and an improvement in glucose metabolism in skeletal muscle (De Matos *et al.* 2018). HIIT is also better at improving insulin sensitivity than moderate continuous training, particularly in those at risk of, or with, T2DM (Jelleyman *et al.* 2015). Together, even though evidence suggests that HIIT might have more metabolic health benefits for women with PCOS than other exercise intensities (Patten *et al.* 2020), studies examining mitochondrial changes with HIIT in women with PCOS are limited.

Training intervention studies in PCOS show diverse findings in relation to mitochondrial characteristics and most of them include moderate-intensity training or a combination of modalities, with only one including HIIT alone (Table 5). One of the studies assessing the effect of an acute exercise bout of 40 min of moderate aerobic exercise in women with PCOS only found an elevated *PGC1A* gene expression within the healthy control group and not in the women with PCOS (Dantas *et al.* 2017). However, the gene expression of *PGC1A* at baseline was higher in the skeletal muscle of women with PCOS compared to controls. This baseline difference could be explained by the fact that IR observed in PCOS could upregulate *PGC1A* expression in an attempt to preserve glucose homeostasis (Dantas *et al.* 2017). Therefore, this exercise bout may only upregulate mitochondrial biogenesis in the healthy controls as *PGC1A* was not already activated by a compensatory mechanism for glucose homeostasis. It is also important to acknowledge that this study did not include a bout of high-intensity exercise but only moderate, and the sample size was small, with only four women in each group. Another study that included a slightly larger sample size (eight women with PCOS and seven healthy controls) examined the prolonged effect of 12 weeks of a combined training protocol (Hutchison *et al.* 2012) of alternating sessions between moderate-intensity continuous training and HIIT. This study reported improvements in cardiorespiratory fitness, BMI and weight, and an increase in insulin sensitivity with training in the PCOS group and a trend for improvement in the control group, with no between-group difference. However, no improvements were observed in any of the groups in gene expression, protein abundance and enzyme activity of mitochondrial markers of biogenesis (PGC1A, TFAM, NRF1), content (CS) and OXPHOS (CIII, CIV) (Hutchison *et al.* 2012). Thus, this may suggest a dissociation of IR from mitochondrial characteristics, which aligns with some studies in T2DM that observed improved IR without changes in mitochondrial function and vice versa (Hey-Mogensen *et al.* 2010, Irving *et al.* 2011). However, more comprehensive studies in women with PCOS are needed to clarify the role of mitochondria in the PCOS-specific IR.

**Table 5:** Effect of exercise interventions on mitochondrial characteristics in skeletal muscle and adipose tissue.

Exercise intervention protocol (study)	Mitochondrial characteristic	Population	Tissue	
			Skeletal muscle	Adipose tissue
**Content**				
(Konopka *et al.* 2015) • 12 weeks (5× 1 h/week) • moderate-intensity AET • stationary bike HR 65% of VO_2_peak	mtDNA copy number	12 obese insulin-resistant PCOS Ex. 13 obese insulin-resistant PCOS Non-Ex.	↔ (within or between groups)	-
	CS activity	12 obese insulin-resistant PCOS Ex. 13 obese insulin-resistant PCOS Non-Ex.	↑ (within PCOS Ex. Group and between PCOS Ex. and PCOS Non-Ex)	-
(Hutchison *et al.* 2012) • 12 weeks (3× 1 h/wk) • moderate- and high-intensity AET; 2-min recovery • treadmill • moderate-intensity: walking/ jogging 70% VO_2_max • high-intensity: 6× 5-min intervals at 95–100% VO_2_max	CS activity	8 obese PCOS Ex. 7 fat mass- & BMI-matched Non-PCOS Ex.	↔ (within and between groups)	-
(Hansen *et al.* 2020) • 14 weeks (3× 45 min/week) • 2 days AET on a bike; high-intensity: 60–65% Wmax for a minimum of 2 min/period • 1 day resistance training: nine different whole-body exercises with three sets of 8–12 reps	CS activity	9 lean insulin-sensitive PCOS Ex. 9 age- & BMI-matched Non-PCOS Ex.	↑ (65% increase within the groups)	-
	CS activity	9 lean insulin-sensitive PCOS Ex. 9 age- & BMI-matched Non-PCOS Ex.	↔ (between the groups)	-
**Respiration-OXPHOS**				
(Hutchison *et al.* 2012) • 12 weeks (3× 1 h/wk) • moderate- and high-intensity AET; 2-min recovery • treadmill • moderate-intensity: walking/jogging 70% VO_2_max • high-intensity: 6× 5-min intervals 95–100% VO_2_max	OXPHOS protein abundance	8 obese PCOS Ex. 7 fat mass- & BMI-matched non-PCOS Ex.	↔ (within and between groups)	-
	Complex III abundance	8 obese PCOS Ex.	↔	-
	Complex IV (sub.4) gene expression	8 obese PCOS Ex.	↑	-
(Konopka *et al.* 2015) • 12 weeks (5× 1 h/wk) • moderate-intensity AET • stationary bike • HR 65% of VO_2_peak	Respiration (state 3)	12 obese insulin-resistant PCOS Ex. 13 obese insulin-resistant PCOS Non-Ex.	↑ (within PCOS Ex.)	-
	Respiration (state 4)	12 obese insulin-resistant PCOS Ex. 13 obese insulin-resistant PCOS Non-Ex.	↔ (within and between groups)	-
	Uncoupled respiration	12 obese insulin-resistant PCOS Ex. 13 obese insulin-resistant PCOS Non-Ex.	↑ (within PCOS Ex.)	-
	Phosphorylation efficiency (ADP:O)	12 obese insulin-resistant PCOS Ex. 13 obese insulin-resistant PCOS Non-Ex.	↑ (within PCOS Ex. and between groups)	-
	Coupling efficiency	12 obese insulin-resistant PCOS Ex. 13 obese insulin-resistant PCOS Non-Ex.	↑ (within PCOS Ex.)	-
(Lionett *et al.* 2021) • 16 weeks (3 days/week) • AET of either low-volume (LV-HIT) or high-volume high-intensity (HV-HIT) but groups were analysed together as a single AET HIIT group • treadmill • (LV-HIT): 10× 1-min intervals at maximal sustainable intensity with 1 min of recovery • (HV-HIT): 4× 4-min intervals at 90–95% HRmax with 3 mins of 70% HRmax recovery	Respiration (state 3)	11 PCOS Ex. 14 age- & BMI-matched Non-PCOS Ex. 7 PCOS Non-Ex.	-	↔ (subcutaneous gluteal) (within or between groups)
	Respiration (state 3)	9 PCOS Ex. 10 age- & BMI-matched Non-PCOS Ex. 7 PCOS Non-Ex.	-	↔ (subcutaneous abdominal) (within or between groups)
**Dynamics**				
(Hutchison *et al.* 2012) • 12 weeks (3 × 1 h/week) • moderate- and high-intensity AET; two min recovery • treadmill • moderate-intensity: walking/jogging 70% VO_2_max • high-intensity: 6x5min intervals 95–100% VO_2_max	*PGC1A* gene expression & protein abundance	8 obese PCOS Ex. 7 fat mass- & BMI-matched Non-PCOS Ex.	↔ (within or between groups)	-
(Dantas *et al.* 2017) • Acute exercise training: 40 min bout • moderate-intensity aerobic exercise 65% VO_2_peak	*PGC1A* gene expression	4 obese PCOS Ex.	↔	-
	*PGC1A* gene expression	4 body composition-matched Non-PCOS Ex.	↑	-
**ROS production**				
(Konopka *et al.* 2015) • 12 weeks (5× 1 h/wk) • moderate-intensity AET • stationary bike • HR 65% of VO_2_peak	mtH_2_O_2_ emissions (during leak/state-4 respiration)	12 obese insulin-resistant PCOS Ex. 13 obese insulin-resistant PCOS Non-Ex.	↓ (within PCOS Ex. and between groups)	-

(↔), no differences; (↓), downregulation; (↑), upregulation; (–), not measured; AET, aerobic exercise training; HR, heart rate; W_max_, maximal Watts; VO_2peak_,peak oxygen uptake; VO_2max_, maximal volume of oxygen uptake; PCOS Ex., women with PCOS allocated in the exercise training group; PCOS Non-Ex., women with PCOS allocated in the sedentary group; non-PCOS Ex., women without PCOS allocated in the exercise training group.

The only study measuring mitochondrial respiration and ROS production in skeletal muscle in women with PCOS included a 12-week moderate-intensity continuous aerobic exercise training (Konopka *et al.* 2015). This study included a lean healthy control group with no intervention and a group of obese insulin-resistant women with PCOS, which were randomised into either the exercise intervention or to a non-exercise group. Following training, women with PCOS exhibited an increase in insulin sensitivity and improvement in mitochondrial respiration and ROS production. The exercise intervention led to increased mitochondrial respiration, maximal CS activity and maximal oxidative capacity and a reduction in mtH_2_O_2_ emissions within the PCOS exercise group. After training, mitochondrial phosphorylation and coupling efficiency and mtH_2_O_2_ emissions were similar between the PCOS exercise group and the lean healthy women (Konopka *et al.* 2015). It is important to note that cardiorespiratory fitness, insulin sensitivity and mitochondrial characteristics were all improved following training in the women with PCOS.

The only study that has examined the effect of HIIT on mitochondrial function was performed in adipose tissue of overweight and lean women with PCOS (Lionett *et al.* 2021). In this study, IR in both women with PCOS and BMI and age-matched controls did not change following HIIT. Consistently, no changes in mitochondrial respiration were detected in subcutaneous abdominal and gluteal adipose tissue in either group after the HIIT intervention (Lionett *et al.* 2021). The outcomes of this study were in contrast to the findings of another study reporting that insulin resistance, fasting insulin levels and glucose infusion rates were all improved after HIIT interventions in women with PCOS (Patten *et al.* 2020). Evidence that exercise training alters insulin sensitivity or mitochondrial characteristics of the adipose tissue even in non-PCOS populations is scarce, and most of the studies have not been able to demonstrate such a response (Dohlmann *et al.* 2018). However, a higher abundance of OXPHOS proteins has been previously observed in adipose tissue of endurance-trained people compared to untrained healthy individuals (Bertholdt *et al.* 2018), as well as an exercise-induced increase in mitochondrial activity and *PGC1A* gene expression in both healthy individuals and patients with T2DM (Dewal & Stanford 2019). Therefore, further studies in women with PCOS are needed to investigate whether exercise training can cause adaptations in mitochondrial characteristics.

A training modality that has had limited research in PCOS is resistance training. To date, only two studies employed resistance training alone in women with PCOS (Almenning *et al.* 2015, Miranda-Furtado *et al.* 2016), while other studies included a combined protocol of both aerobic and resistance training (Bruner *et al.* 2006, Thomson *et al.* 2008, Hansen *et al.* 2020). None of the studies with resistance training alone investigated mitochondrial characteristics, and only Almenning and colleagues assessed IR showing improvements in HOMA-IR in women with PCOS after the intervention. Of the three combined training interventions studies including both HIIT and resistance training, two did not measure IR but reported significant decreases in fasting insulin levels in women with PCOS (Bruner *et al.* 2006, Thomson *et al.* 2008), while Hansen *et al.* showed improved insulin sensitivity (euglycaemic–hyperinsulinaemic clamp) in healthy women without PCOS, but not in lean women with PCOS (Hansen *et al.* 2020). In this study, the only mitochondrial characteristic measured in skeletal muscle was CS activity, which was increased by 65% in both women with and without PCOS after the 14-week intervention with no significant differences between groups (Hansen *et al.* 2020). Thus, this suggests a lack of direct link between increased mitochondrial function and whole-body insulin sensitivity.

No studies have investigated the effect of exercise training on mitochondrial dynamics in any tissue from women with PCOS. A previous study in people who are obese reported that an acute exercise bout might enhance mitophagy and may alter the expression of mitochondrial fusion and fission proteins, promoting the mitochondrial network for future bouts (Axelrod *et al.* 2019). In contrast, exercise training may reduce mitophagy by improving the integrity of the mitochondrial network or by increasing the size and abundance of intact mitochondrial networks (Axelrod *et al.* 2019). However, due to numerous confounding variables it is difficult to extrapolate these findings to women with PCOS, and therefore, future research is necessary to elucidate the effects of exercise on mitochondrial dynamics in these women.

## Conclusion

PCOS is a complex endocrine disorder with heterogeneous clinical manifestations. Despite the high prevalence of IR in this complex multifaceted disorder, the existence of mitochondrial dysregulation and its potential role in the pathogenesis of PCOS is not clear.

Studies in skeletal muscle consistently detect no differences between PCOS and control groups in terms of mitochondrial content, but none to-date have used the gold-standard measure of transmission electron microscopy. Findings on mitochondrial respiration, OXPHOS and biogenesis in skeletal muscle are contradictory, and studies have yet to investigate mitochondrial dynamics in this tissue. Evidence in adipose tissue shows decreased mitochondrial respiration in the abdominal adipose tissue in women with PCOS; however, evidence is limited to one study.

To date, most exercise interventions are suggested to benefit mitochondrial health in skeletal muscle of women with PCOS by enhancing some respiration states and decreasing ROS production along with improving insulin sensitivity. Besides the fact that some exercise training did improve insulin sensitivity, this did not seem to improve mitochondrial content or biogenesis markers, and findings in OXPHOS complexes expression are contradictory. Current data in adipose tissue are limited to one study showing no changes in mitochondrial respiration. Therefore, further research examining exercise-induced mitochondrial changes is needed in this tissue.

Remarkably, despite exercise training studies in women with PCOS improving cardiorespiratory fitness, insulin sensitivity, HOMA-IR and body composition, this might not cause an improvement in mitochondrial characteristics. This indicates that mitochondrial function might not be linked to IR in PCOS and further comprehensive investigations are required. In addition to that, the majority of current intervention studies in PCOS did not assess HIIT alone in skeletal muscle, which is generally known to improve mitochondrial function. Therefore, further evidence is needed to elucidate the impact of exercise training and intensities, and in particular HIIT, on mitochondria profile in these tissues, and whether these mitochondrial changes are responsible for the exercise-induced improvements of IR in women with PCOS.

Taken together, current studies are limited to confirming the presence of an existing tissue-specific mitochondrial dysregulation in PCOS, independent of obesity, and the role this plays in IR. Further comprehensive large-scale exercise intervention studies are required to understand the association between metabolic dysfunction and aberrant mitochondrial profile and the molecular mechanisms underlying the exercise-induced metabolic adaptations in PCOS. These findings may ultimately contribute to improving the metabolic, reproductive and mental health of women with PCOS and may help identify new therapeutic approaches for the management of this syndrome.

## Declaration of interest

The authors declare that there is no conflict of interest that could be perceived as prejudicing the impartiality of this review.

## Funding

This work did not receive any specific grant from any funding agency in the public, commercial or not-for-profit sector.
